# Heat of Formation of Calcium Aluminate Monosulfate at 25 °C

**DOI:** 10.6028/jres.067A.001

**Published:** 1963-02-01

**Authors:** H. A. Berman, E. S. Newman

## Abstract

The heat of formation of calcium aluminate monosulfate, 3CaO·Al_2_O_3_·CaSO_4_·12H_2_O, at 25 °C, and of less completely hydrated samples of the same compound, was determined by the heat-of-solution method, with 2*N* HCl as the solvent, and 3CaO·Al_2_O_3_·6H_2_O(c) and CaSO_4_·2H_2_O(c), as the reactants. The results were as follows:
Δ*H, kj/mole*Δ*H, kcal/mole*3CaO·Al_2_O_3_·CaSO_4_·12H_2_O(c) Heat of formation  from elements, 
$\Delta H_{f}^{{}^{\circ}}$−2100  from reactants and H_2_O(1)−15.0 Heat of solution in 2*N* HCl− 495.7− 118.5 Change of heat of solution  with H_2_O content at 12H_2_O, per mole H_2_O
$\frac{d\left(\Delta H\right)}{dn}$1.93

The heat of the reaction (Δ*H*)

$$\begin{array}{r} 3\mathrm{CaO}\cdot {\mathrm{Al}}_{2}O_{3}\cdot {\mathrm{CaSO}}_{4}\cdot 12H_{2}O\left(c\right)+2\left({\mathrm{CaSO}}_{4}\cdot 2H_{2}O\right)\left(c\right)+15H_{2}O\left(l\right)\to \\ 3\mathrm{CaO}\cdot {\mathrm{Al}}_{2}O_{3}\cdot 3{\mathrm{CaSO}}_{4}\cdot 31H_{2}O\left(c\right) \end{array}$$

is −134.4 kj/mole or −32.1 kcal/mole. The heat of the reaction (Δ*H*)

$$\begin{array}{r} 3\mathrm{CaO}\cdot {\mathrm{Al}}_{2}O_{3}\cdot {\mathrm{CaSO}}_{4}\cdot 12H_{2}O\left(c\right)+2\left({\mathrm{CaSO}}_{4}\cdot 2H_{2}O\right)\left(c\right)+16H_{2}O\left(l\right)\to \\ 3\mathrm{CaO}\cdot {\mathrm{Al}}_{2}O_{3}\cdot 3{\mathrm{CaSO}}_{4}\cdot 32H_{2}O\left(c\right) \end{array}$$

is −144.9 kj/mole or −34.6 kcal/mole.

Values reported earlier for the heat of formation of calcium aluminate trisulfate and of calcium aluminate monocarbonate should be revised by adding −0.9 kcal/mole to each reported Δ*H* value, with the following resulting values:
Δ*H* from appropriate reactants
$\Delta H_{f}^{{}^{\circ}}$*kcal/mole**kcal/mole*3CaO·Al_2_O_3_·3CaSO_4_·31H_2_O(c) −47.01 −41233CaO·Al_2_O_3_·3CaSO_4_·32H_2_O(c) −49.52 −41943CaO·Al_2_O_3_·CaCO_3_·10·68H_2_O(c) −19.77 −1957

Conditions for the formation of the monosulfate from solution, and its properties on exposure to moisture, are discussed.

## 1. Introduction

Calcium aluminate monosulfate, 3CaO·Al_2_O_3_·CaSO_4_·12H_2_O, also referred to as calcium monosulfoaluminate, is one of two complex salts that may be formed by the aggressive action of sulfate waters on portland cement. It is also an intermediate or a final product in the hydration of most portland cements, derived from the gypsum and tricalcium aluminate which they contain in the anhydrous state. Depending on the availability of the various reacting compounds, either the monosulfate or calcium aluminate trisulfate, 3CaO·Al_2_O_3_·3CaSO_4_· 31–33H_2_O, may be formed [1, 2].^1^

The aluminate sulfates are representative, respectively, of two series of complex calcium aluminates, which may be represented by the general formulas 3CaO·Al_2_O_3_·Ca*X*·*n*H_2_O and 3CaO·Al_2_O_3_·3Ca*X*·*m*H_2_O, where *X* is a divalent ion or two units of a monovalent ion, *n* is 10 to 12, and *m* is approximately 32. As with many compounds encountered in portland cement chemistry, the water content is not always definite. Some of the water in these compounds is tightly bound chemically; the remainder is more loosely bound and capable of variation with the ambient temperature or humidity.

As part of a continuing investigation of the thermochemical properties of substances occurring in hydraulic cements and their reaction products, the heat of formation of hydrated calcium aluminate monosulfate has been determined.

Measurements by the heat-of-solution method were made of the heat evolved at 25 °C in the reaction

$$\begin{array}{c} 3\mathrm{CaO}\cdot {\mathrm{Al}}_{2}O_{3}\cdot 6H_{2}O\left(c\right)+{\mathrm{CaSO}}_{4}\cdot 2H_{2}O\left(c\right)+4H_{2}O\left(l\right)\\ \overset{\Delta H_{1}}{\to }3\mathrm{CaO}\cdot {\mathrm{Al}}_{2}O_{3}\cdot {\mathrm{CaSO}}_{4}\cdot 12H_{2}O\left(c\right) \end{array}$$ (1)

The heat of this reaction is the difference between the sum of the heats of solution of the reactants and the heat of solution of the product, in accordance with the following equations:

$$3\mathrm{CaO}\cdot {\mathrm{Al}}_{2}O_{3}\cdot 6H_{2}O\left(c\right)+12\mathrm{HCl}\left(\mathrm{aq}\right)\overset{\Delta H_{2}}{\to }\left[3{\mathrm{CaCl}}_{2}+2{\mathrm{AlCl}}_{3}+12H_{2}O\right]\left(\mathrm{aq}\right)$$ (2)

$${\mathrm{CaSO}}_{4}\cdot 2H_{2}O\left(c\right)+\left[3{\mathrm{CaCl}}_{2}+2{\mathrm{AlCl}}_{3}+12H_{2}O\right]\left(\mathrm{aq}\right)\overset{\Delta H_{3}}{\to }\left[3{\mathrm{CaCl}}_{2}+2{\mathrm{AlCl}}_{3}+{\mathrm{CaSO}}_{4}+14H_{2}O\right]\left(\mathrm{aq}\right)$$ (3)

$$\begin{array}{c} 4H_{2}O\left(1\right)+\left[3{\mathrm{CaCl}}_{2}+2{\mathrm{AlCl}}_{3}+{\mathrm{CaSO}}_{4}+14H_{2}O\right]\left(\mathrm{aq}\right)\overset{\Delta H_{4}}{\to }\\ \left[3{\mathrm{CaCl}}_{2}+2{\mathrm{AlCl}}_{3}+{\mathrm{CaSO}}_{4}+18H_{2}O\right]\left(\mathrm{aq}\right) \end{array}$$ (4)

$$\begin{array}{c} 3\mathrm{CaO}\cdot {\mathrm{Al}}_{2}O_{3}\cdot {\mathrm{CaSO}}_{4}\cdot 12H_{2}O\left(c\right)+12\mathrm{HCl}\left(\mathrm{aq}\right)\overset{\Delta H_{5}}{\to }\\ \left[3{\mathrm{CaCl}}_{2}+2{\mathrm{AlCl}}_{3}+{\mathrm{CaSO}}_{4}+18H_{2}O\right]\left(\mathrm{aq}\right) \end{array}$$ (5)

The summation, equation 2+equation 3+equation 4−equation 5, results in equation 1. Similar summation of the Δ*H* values results in Δ*H*_1_.

The heat of solution of each of the reactants and of the product was measured in HCl·26·61H_2_O (2.00*N* HCl at 25 °C). The heats of formation of the reactants were taken from Circular 500 [3] of the National Bureau of Standards.

All calculations in this paper are based on the 1961 atomic weight table [4] and on the thermochemical calorie, defined as exactly 4.184 joules. Differences between the 1961 and 1957 atomic weight tables are insignificant for this work, and the results obtained are therefore consistent with work published earlier on calcium aluminate monocarbonate [5] and calcium aluminate trisulfate [6], except for the revisions to this earlier work noted in sections 4.5 and 4.7.

## 2. Preparation and Analysis of Samples

### 2.1. Preparation

The calcium aluminate sulfates can be formed as white precipitates when aqueous solutions of calcium hydroxide, sulfate ion, and of compounds containing aluminum ion or aluminate ion are mixed.

At room temperature the trisulfate is the eventual product in all aqueous concentration ranges studied. Within a limited concentration range, however, the monosulfate can be formed first and may be isolated by filtration before conversion to the trisulfate takes place. The conditions for formation of the monosulfate and for producing the compound in adequate quantity are discussed in the appendix to this paper.

Eight preparations of calcium aluminate monosulfate were made. Details of the reaction mixtures and of the products obtained are given in table 1, together with details of some mixtures in which the trisulfate was formed. Preparations 1, 2, 3, 4, and 5 were made from saturated Ca(OH)_2_ solution and calcium aluminate solution. The yields obtained in these batches were 9, 6, 7, 5, 11 g, respectively. Preparations 6, 7, and 8 were made from Al_2_(SO_4_)_3_ solution and saturated Ca(OH)_2_ solution by the alternate reaction-decantation technique described in the appendix, a technique which makes a larger yield of the monosulfate possible in the equipment available and within the time limits necessary to prevent conversion of the monosulfate to the trisulfate. Yields of batches 6, 7, and 8 were 16, 30, and 33 g respectively. The quantities obtained were about 75 percent of the theoretical yield, largely as a result of mechanical loss in the handling operations.

Preparation of the reagents, transfer of solutions, mixing, filtration, and other operations were performed in closed systems with precautions taken to exclude CO_2_. Solutions were made with distilled water which had been boiled and then cooled in a current of CO_2_-free air.

All batches were dried at 33 percent relative humidity in a desiccator over saturated MgCl_2_·6H_2_O [7], except sample 8 which was dried at 12 percent relative humidity (saturated LiCl) [7]. Samples conditioned in this way are referred to hereinafter as “original” samples. Several portions of samples 2, 6, 7, and 8 were dried at 12 percent relative humidity, 5 percent relative humidity (23*N* sulfuric acid) [8], and 0 percent relative humidity (CaO). These samples are referred to as “dried” samples. Before placing the samples in the desiccators and after opening them at any time, the desiccators were evacuated to 2 to 4 cm Hg pressure and refilled with CO_2_-free air which had been passed through the same conditioning solution as contained in the desiccator. Inasmuch as effective conditioning requires slow air flow, the desiccators were generally opened only once during a day, evacuated rapidly, and refilled overnight with conditioned air; thus, they were generally under vacuum most of this period. The desiccator containing CaO was kept under vacuum at all times, except when it was opened to remove a sample. When the loss on ignition of a preparation was constant, the material was ground and mixed in a sealed glass jar containing wood balls, and the ground material was stored in the appropriate desiccator.

Heat-of-solution determinations were made on all the “original” and “dried” preparations. Measurements were also made of the heats of solution of portions of sample 7 exposed for various periods of time to an atmosphere at 100 percent relative humidity. The latter environment was obtained by slowly passing dry CO_2_-free air through CO_2_-free distilled water and into a desiccator containing the samples to be exposed. The air was passed through water in two spiral gas-washing bottles and then through a delivery tube fitted with a fritted-glass opening immersed in a reservoir of water in the desiccator.

### 2.2. X-ray Diffraction and Chemical Analysis

X-ray diffraction patterns of the preparations were obtained by the powder method on a Geiger-counter diffractometer with copper K*α* radiation. The principal peaks of the patterns are summarized in table 2. The samples conditioned at 33 percent relative humidity showed peaks of both the high- and low-humidity forms of the compound [9], except in the case of sample 3, which showed only the peaks of the high-humidity form at 8.8 and 4.4 A. There was some indication of a transformation of samples while on the machine, quick patterns sometimes showing a higher intensity of the 8.8 and 4.4 A peaks compared to the 8.2 and 4.0 peaks, respectively, of the low-humidity form, than long-term patterns run later on the same specimen. There is reason to believe, therefore, that samples taken for calorimetry contained more of the high-humidity form than the X-ray patterns indicate. Wet samples taken immediately after filtration showed the pattern characteristic of “wet-slice” calcium aluminate monosulfate [9] with the most intense lines at 9.8 and 4.9 A and an intermediate weaker line at 5.7 A. This pattern is consistently different in intensity relationships from the calcium aluminate trisulfate pattern, which has its most intense lines at 9.8 and 5.6 A and a weaker line at 5.0 A.

The oxide analyses of the preparations are given in table 1 and were obtained by replicate (2 to 5) measurements, except in the case of sample 1, for which material was available for only one measurement. The precision of the analyses and of the calculated mole ratios of the oxides (and of compounds calculated from the oxides) was determined. The estimates of standard error^2^ for each oxide determination were calculated separately for each preparation, rather than by pooling the standard- error estimates for that particular determination for all preparations as had been done in earlier work [6]. This procedure is based on the assumption that, for a particular preparation, the calculated precision of the analysis is dependent on the uniformity of the preparation and not simply on the degree to which an analytical method can be reproduced. The calculated precision of the mole ratios of the compounds assumed to be present as impurities is an indication of the quantitative reliability of the impurity calculations. The designation of the compounds present as impurities was based on the chemical analysis, optical microscopic examination, and X-ray diffraction patterns.

The preparation of the gypsum and of the hydrated tricalcium aluminate has been discussed in reference [6].

## 3. Heat-of-Solution Measurements

The heats of solution were determined in HC1·26.61_4_ H_2_O (2.00*N* HCl at 25 °C). The improved all-platinum calorimeter described in an earlier publication [5] was used, with platinum resistance thermometer, air jacket, and water bath controlled to ±0.005 °C or better. The calorimeter was stirred with the shorter of the two stirrers described, a 4-in. platinum stirrer with four-bladed propeller. Samples were introduced through a glass funnel. When the calorimeter temperature attained after the sample has dissolved is close to the temperature of the bath and the room, corrections for the heat capacity of the sample are minimized by the funnel-introduction technique.

Since it was inconvenient to prepare the calcium aluminate monosulfate in large quantities, 1-g samples of the compound were used with the normal quantity of acid (740 g) for the calorimeter. The quantities of 3CaO·Al_2_O_3_·6H_2_O and CaSO_4_·2H_2_O required by the stoichiometry of eqs (1) to (5) are 0.61 and 0.28 g respectively (in 740 g HCl) corresponding to 0.49 and 0.22 g in 600 g HCl. The heat of solution of 3CaO·Al_2_O_3_·6H_2_O was determined in earlier work [5, 6] in a platinum-lined calorimeter described by Newman [10] with samples ranging from 0.26 to 0.44 g in 600 g HCl, a sample-acid ratio slightly below the ratio required for this work. It has been shown, however, that there is no observable heat-of-dilution effect for samples of 3CaO·Al_2_O_3_·6H_2_O in 2*N* HCl between 0.26 and 2.9 g [5, 11]. The determinations with the 0.26 to 0.44-g samples were therefore used for this work.

The heat of solution of 0.28 g of CaSO_4_·2H_2_O in 740 g of 2*N* HCl was separately determined for calculating the heat of reaction (eq (1)), in preference to the results of earlier determinations obtained with sample-acid ratios equivalent to 1.2 g and 0.53 g of CaSO_4_·2H_2_O in 740 g of acid. The heats of solution obtained with the 0.28-g samples were used because they were slightly higher than for the larger samples, but the differences (see sec.4.1) are not considered sufficiently significant to suppose that a heat-of- dilution effect was actually observed. Although, according to eq (3), the gypsum should be added to the acid solution obtained from the dissolution of the tricalcium aluminate, it was actually added to the fresh acid. As has been shown [6], no measurable error is thereby introduced.

The heat effect of adding the 4 moles of H_2_O appearing in eqs (1) and (4) was estimated as the partial molal heat content of H_2_O in 2*N* HCl [3], neglecting the contribution of the small amounts of other solutes present.

## 4. Results and Discussion

### 4.1. Heats of Solution of the Preparations

The heats of solution obtained with the original preparations (samples 1–8) are shown in table 1. Those obtained with the dried preparations are shown in table 3. Both tables also show heats of solution of the pure calcium aluminate monosulfate, after correction for the impurities in the samples.

The heat capacities obtained in the calorimeter calibrations and the data from which these heat capacities were calculated, are listed in table 4. Detailed data from the heat-of-solution determinations are shown in table 5.

Determinations of the heat of solution of CaSO_4_·2H_2_O are shown in table 6, for sample-acid ratios of 0.0004, 0.0007, and 0.0017. For a range of ±1 standard error the results for the lowest sample- acid ratio do not quite overlap those of the other two sets, but the gap is not great enough to be statistically significant. Perhaps a more precise determination of the heat of solution of CaSO_4_·2H_2_O at different sample-acid ratios would reveal a significant heat-of-dilution effect. In any event, the heat of solution obtained for the lowest ratio has been used, for the reason discussed in section 3.

### 4.2. Correction for Impurities

The calculation of the composition of a sample and of the heat of solution of the pure calcium aluminate monosulfate present was performed as described in the appendices to references [5] and [6].

In general, X-ray diffraction patterns were useful only in revealing the major components, whereas optical microscopic examination was generally necessary to confirm the impurities suggested by the chemical analysis. In the case of samples exposed at 100 percent relative humidity, the X-ray pattern indicated breakdown of the aluminate monosulfate into compounds not detectable by chemical analysis (see sec.4.8). Another useful indication of the nature of the impurities, also described in section 4.8, was the pattern of evolution of heat during the dissolution of the sample in the calorimeter.

As in earlier work on the heat of formation of calcium aluminate trisulfate [6], the presence of a small quantity of CO_2_ not offset by CaO was a troublesome factor. (Note that in sample 6 the standard error of the free CO_2_ was considerably smaller than the quantity of free CO_2_ calculated, indicating a high confidence in its presence, whereas in sample 7 the standard error was almost as large as the quantity itself, indicating the reverse). This free CO_2_, if actually present, is assumed to be sorbed much like free H_2_O, and the quantity of heat it would liberate or absorb on dissolution of the compound is neglected. In reference [6], the heat of solution of hydrated alumina was also neglected, because this compound remained in suspension and did not contribute to the total heat evolved by the preparation. However, in these monosulfate preparations, clear solutions were obtained in the calorimeter, indicating that the hydrated alumina did dissolve (see sec.4.7 for exceptions). Correction was therefore made for its heat of solution. Assarsson [12] points out that soluble hydrated alumina, obtained from precipitates that have digested for only a short time, contains between 3 and 4 moles of H_2_O, and suggests that the additional H_2_O is physically adsorbed on a particle that is chemically gibbsite, A1_2_O_3_·3H_2_O. Correction for the heat of solution of the hydrated alumina is therefore calculated as a gibbsite correction. In samples 1, 2, 4, and 5, the uncertainty in the H_2_O content of the gibbsite brings about a corresponding uncertainty in the H_2_O content of the aluminate monosulfate: if the alumina is Al_2_O_3_·4H_2_O, this uncertainty ranges from 0.01 to 0.04 moles per mole monosulfate. The effect of this uncertainty on the heat of solution of the monosulfate is, however, only 1 part in 10,000 for 3CaO·Al_2_O_3_·CaSO_4_·12H_2_O.

**Figure 1:**
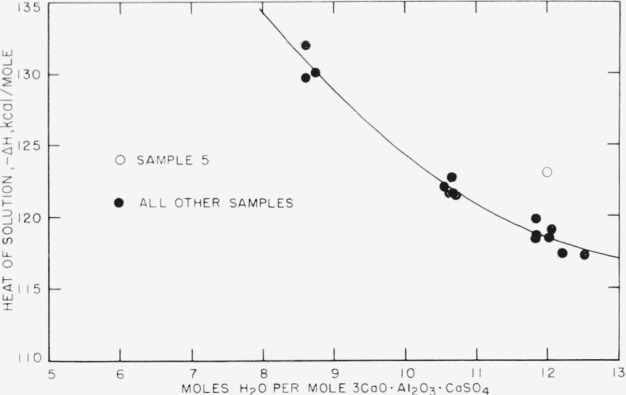
*Heat of solution of* 3CaO·Al_2_O_3_·CaSO_4_·*n*H_2_O(*c*)

The mixture in which sample 5 was precipitated was on the border of the concentration range for formation of the monosulfate, under the experimental conditions of this work (see appendix, 7.1). Microscopical examination showed needles of calcium aluminate trisulfate with negative elongation, together with the usual needlelike “fibers” of the monosulfate which appear to have positive elongation because they are uniaxial negative plates observed edgewise. Since both the monosulfate and the trisulfate have variable water contents, it was necessary to assume a distribution of H_2_O molecules between the two. The H_2_O balance most nearly corresponding to the analytical data was obtained by assuming 12H_2_O for the monosulfate and 31H_2_O for the trisulfate. The compound composition and corrected heat of solution shown in table 1 were then calculated. However, the point in figure 1 corresponding to this sample (open circle) shows the greatest deviation from the curve. Because of the uncertainties in the calculation of H_2_O content and of other compounds and impurities for sample 5, this point was not included in establishing the curve.

Insufficient quantities of samples 1 and 4 made it necessary to assume water contents by subtraction of the total percentage of CaO, Al_2_O_3_, SO_3_, and CO_2_ from 100 percent. The CO_2_ content of sample 4 was not determined, but was estimated from that of the other samples instead. Only one determination of major oxides could be made on sample 1. Although it was not possible, for this reason, to calculate standard errors for these analyses, the uncertainties in the final calculation of heat of solution and water content are not believed to be very great for these two samples. If the points in figure 1 corresponding to these samples were omitted, there would be almost no change in the curve.

The following values were used for the heats of solution of the various impurities:
3CaO · Al_2_O_3_ · 3CaSO_4_ · 31H_2_O−Δ*H* =        74.94 kcal/mole [6]CaCO_3_83.61 cal/g [5]Ca(OH)_2_436.41 cal/g [13]CaSO_4_·2H_2_O          −33.81 cal/g from table 6CaSO_4_·l/2H_2_O−7.64 cal/g [14]CaSO_4_ (soluble anhydrite) 10.31 cal/g [14]Al_2_O_3_·3H_2_O 50.34 kcal/mole

The value for the heat of solution of Al_2_O_3_·3H_2_O was derived by calculation from the heats of formation of gibbsite (hydrargyllite), −Δ*H*=613.7 kcal/mole [3]; *α*-Al_2_O_3_ (corundum), 399.09 kcal/mole [3]; 3H_2_O, 204.95 kcal/mole [3]; and the heat of solution of *α*-Al_2_O_3_ in 2.00*N* HCl, 59.95 kcal/mole [15]; with a small correction for the heat of dilution of HCl with the H_2_O, estimated to be 0.05 kcal.

### 4.3. Heat of Solution of Calcium Aluminate Monosulfate

Figure 1 is a plot of the corrected heats of solution against the calculated water content of the pure compound. The points are derived from tables 1 and 3. The solid curve represents the following least-squares quadratic equation calculated from the points:

$$-\Delta H=214.664-14.096n+0.5069n^{2}$$ (6)

where

Δ*H*: = heat of solution in kcal/mole calcium aluminate monosulfate
*n*: = moles H_2_O/mole calcium aluminate monosulfate

This curve covers the range of 8 to 13H_2_O; that is, the samples conditioned at relative humidities of 33 percent and below. For reasons discussed in the preceding section, sample 5 (open circle) was omitted in calculating the equation. For the samples conditioned at 100 percent relative humidity, with water contents greater than 13H_2_O, see the discussion in section 4.8.

No correlation was apparent between the heats of solution and the various X-ray patterns characteristic of the monosulfate, except to the extent that the pattern showing the 9.0 and 4.5 A peaks was more prominent in the 33 percent relative-humidity- conditioned samples, which had the lower heat-of- solution values, and the 8.0 and 4.0 A peaks were more prominent in the dried samples, which had the higher heats of solution. One sample, No.3, conditioned at 33 percent relative humidity, showed only the 9.0–4.5 A pair; one, No.6 at 0 percent relative humidity, showed only the 8.0–4.0 A pair. All other patterns obtained (see table 2) were mixtures of the two basic patterns. There was no observable relation between the relative heights of the respective peaks and the heats of solution. However, the correlation between water content and heat of solution is definite. Since the X-ray specimens were not protected against room atmosphere while mounted on the diffractometer, it is uncertain whether the variation of heat of solution with water content is a function of water content alone or of the crystal-structure change reflected by the change in X-ray pattern as well.

From eq (6), the heat of solution of 3CaO·Al_2_O_3_·CaSO_4_·12H_2_O in 2.00*N* HCl (at a sample-acid ratio of 1:740) is −ΔH = 118.5 kcal/mole. The change of the heat of solution with water content at this point, *d*(−Δ*H*)/*dn*, is −1.93 kcal/mole per mole H_2_O.

Corresponding values for the heats of solution of compositions containing less H_2_O have been calculated from eq (6) and assembled in table 7.

### 4.4. Heat of Formation of the Product from the Reactants

The heat of the reaction represented by eq (1) is calculated from the heats of solution of the reactants and products. The heat effects of eqs (2), (3), (4), and (5) are added, as follows: (Note, however, that the tricalcium aluminate Hydrate used as one of the reactants was actually 3CaO·Al_2_O_3_·5.859H_2_O [6] and a further correction must be made later to correct for this departure from eq (2)). For
eq (2),−Δ*H*=139.163 kcal/mole     (3)   −5.281     (4)    + 0.066     (5)  −118.506     (1)+Δ*H*= 15.44

### 4.5. Correction for the Water Content of the Tricalcium Aluminate

The heat of solution of hydrated tricalcium aluminate was measured on a sample containing 5.859H_2_O instead of 6H_2_O. It is therefore necessary to introduce a correction to the summation in section 4.4 based on the heat effect of the following equation:

$$3\mathrm{CaO}\cdot {\mathrm{Al}}_{2}O_{3}\cdot 6H_{2}O\left(c\right)\to 3\mathrm{CaO}\cdot {\mathrm{Al}}_{2}O_{3}\cdot 5.859H_{2}O\left(c\right)+0.141H_{2}O\left(l\right)$$ (7)

In references [5, 6], the heat of this reaction was taken as 0.141/6.00 times the heat of hydration of anhydrous 3CaO·Al_2_O_3_ to the hexahydrate. This estimate was based on the assumption that the fraction hydrated to 6H_2_O was 5.859/6.00, and that the remainder was anhydrous. From the measurements of Thorvaldson, Brown, and Peaker [11], the heat effect was calculated to be −Δ*H*= −1.36 kcal/mole 3CaO·Al_2_O_3_. At this time, it seems to the authors more reasonable to adopt a figure for the heat effect of eq (7) derived from a continuous plot of the heat of formation of several hydrates of tricalcium aluminate against their water content. Data for such a plot between 0 and 11.6H_2_O are also available from Thorvaldson, Brown, and Peaker’s paper [11]. The points lie on a continuous curve, from which the heat effect of eq (7) may be estimated as −Δ*H*=−0.46 kcal/mole, about one-third the figure previously used for this correction [5, 6].

If the heat of reaction calculated in section 4.4 is corrected for the heat effect of eq (7), the heat of reaction for eq (1) is then −Δ*H*=15.44 −0.46=14.98 kcal/mole.

Heats of formation from the same reactants of compositions containing less H_2_O, calculated in the same manner, are given in table 7.

### 4.6. Heat of Formation of Calcium Aluminate Monosulfate from the Elements

The heat of formation of calcium aluminate monosulfate is the sum of the heat effect of eq (1) and of the heats of formation of the reactants:
*kcal/mole*.
$\Delta H_{f}^{{}^{\circ}}$ 3CaO·Al_2_O_3_·6H_2_O(c)= −1329.
$\Delta H_{f}^{{}^{\circ}}$ CaSO_4_·2H_2_O(c)= −  483.06
$\Delta H_{f}^{{}^{\circ}}$ 4H_2_O(1)= −  273.27Δ*H*
eq (1)= −    14.98
$\Delta H_{f}^{{}^{\circ}}\;3\mathrm{CaO}\cdot {\mathrm{Al}}_{2}O_{3}\cdot {\mathrm{CaSO}}_{4}\cdot 12H_{2}O\left(c\right)=-2100_{.31}$

**Figure 2:**
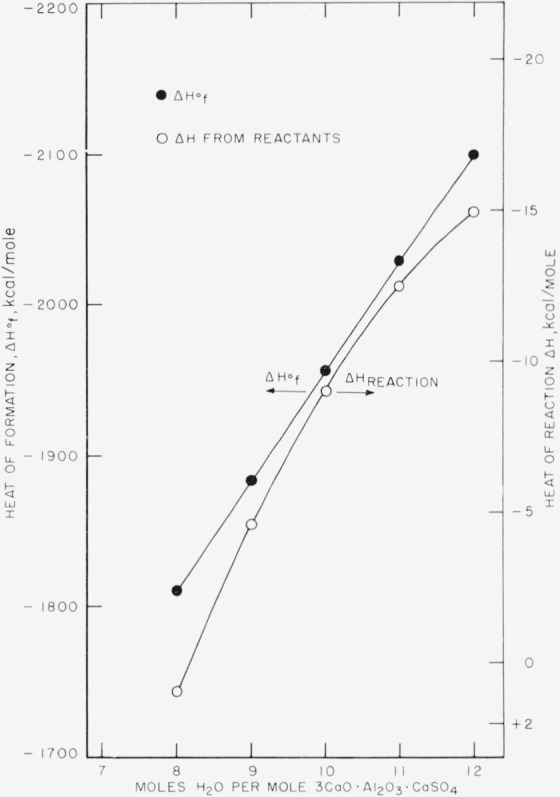
Heat of formation of *3CaO·Al_2_O_3_·CaSO_4_*·n*H_2_O(*c**) Closed circles: $\Delta H_{f}^{{}^{\circ}}$ (from elements). Open circles: Δ*H* from reactants 3CaO·Al_2_O_3_·6H_2_O(c), CaSO_4_·2H_2_O(c), and H_2_O(l).

The heats of formation of compositions with water contents less than 12H_2_O, similarly calculated, are shown in table 7 and figure 2. The heat effect of introducing H_2_O into the compound at any stage of hydration can be determined from this figure.

### 4.7. Other Heats of Reaction

The revised correction for the heat of solution of tricalcium aluminate hexahydrate, described in section 4.5, must be applied to the heats of formation of calcium aluminate trisulfate and calcium aluminate monocarbonate, reported by the authors in references [5, 6]. To each of the reported values for Δ*H_f_*, it is necessary to add the difference between 0.46 and 1.36, or −0.9 kcal/mole. The following revised values for the heats of formation are then obtained:
Δ*H* from reactants^3^ kcal/mole
$\Delta H_{f}^{{}^{\circ}}$ kcal/mole3CaO·Al_2_O_3_·3CaSO_4_·31H_2_O(c)−47.01−41233CaO·Al_2_O_3_·3CaSO_4_·32H_2_O(c)−49.52−41943CaO·Al_2_O_3_·CaCO_3_·10.68H_2_O(c)−19.77−1957

The heat effects in the stepwise formation of the two complex calcium aluminate sulfates may be calculated from their heats of formation and summarized as follows:

The heat of the reaction

$$\begin{array}{c} 3\mathrm{CaO}\cdot {\mathrm{Al}}_{2}O_{3}\cdot 6H_{2}O\left(c\right)+{\mathrm{CaSO}}_{4}\cdot 2H_{2}O\left(c\right)+4H_{2}O\left(1\right)\to \\ 3\mathrm{CaO}\cdot {\mathrm{Al}}_{2}O_{3}\cdot {\mathrm{CaSO}}_{4}\cdot 12H_{2}O\left(c\right) \end{array}$$ (1)

Is

$$\Delta H=-14.9_{8}\;\mathrm{kcal}/\mathrm{mole}$$

From the heats of reaction of 3CaO·Al_2_O_3_·6H_2_O(c), CaSO_4_·2H_2_O(c), and H_2_O(1) to both the monosulfate (eq (1)) and the trisulfate (section 4.7 and ref. [6]), the heat of the reaction

$$\begin{array}{c} 3\mathrm{CaO}\cdot {\mathrm{Al}}_{2}O_{3}\cdot {\mathrm{CaSO}}_{4}\cdot 12H_{2}O\left(c\right)\\ +2\left({\mathrm{CaSO}}_{4}\cdot 2H_{2}O\right)\left(c\right)+15H_{2}O\left(l\right)\to \\ 3\mathrm{CaO}\cdot {\mathrm{Al}}_{2}O_{3}\cdot 3{\mathrm{CaSO}}_{4}\cdot 31H_{2}O\left(c\right) \end{array}$$ (8)

is

$$\Delta H=-32.0_{3}\;\mathrm{kcal}/\mathrm{mole}$$

and for the reaction

$$\begin{array}{c} 3\mathrm{CaO}\cdot {\mathrm{Al}}_{2}O_{3}\cdot {\mathrm{CaSO}}_{4}\cdot 12H_{2}O\left(c\right)\\ +2\left({\mathrm{CaSO}}_{4}\cdot 2H_{2}O\right)\left(c\right)+16H_{2}O\left(l\right)\to \\ 3\mathrm{CaO}\cdot {\mathrm{Al}}_{2}O_{3}\cdot 3{\mathrm{CaSO}}_{4}\cdot 32H_{2}O\left(c\right) \end{array}$$ (9)

is

$$\Delta H=-34.5_{4}\;\mathrm{kcal}/\mathrm{mole}.$$

### 4.8. Effect of Exposure of Calcium Aluminate Monosulfate to Moisture

**Figure 3:**
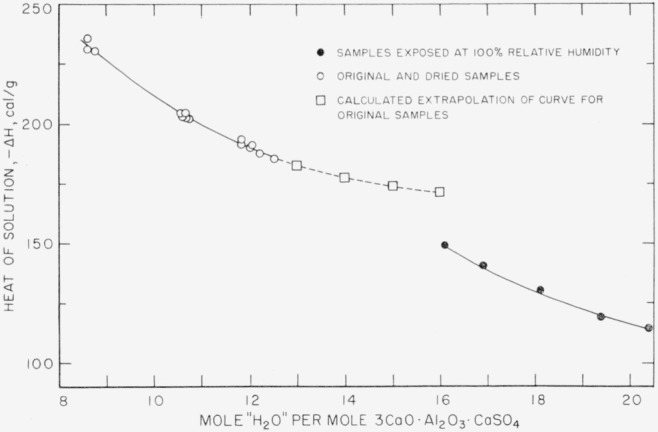
Heat of solution of calcium aluminate monosulfate samples exposed to 100 percent relative humidity, compared to that of the original and the dried preparations.

Attempts to examine the effect of H_2_O contents greater than 12.5H_2_O on the heat of solution of the compound were not successful. Table 5 shows the heats of solution (in cal/g) and nominal water contents of portions of sample 7 exposed to 100 percent relative humidity for various lengths of time up to 2 months. If these values are plotted together with the corresponding values for the original and dried samples, the curve for the moist samples is not continuous with the curve for the others (fig.3).

It is believed that the discontinuity observed is due to chemical decomposition of the monosulfate. Evidence obtained from X-ray diffraction data, chemical tests, and calorimetric observations suggests the following reaction:

$$\begin{array}{c} 3\left(3\mathrm{CaO}\cdot {\mathrm{Al}}_{2}O_{3}\cdot {\mathrm{CaSO}}_{4}\cdot 12H_{2}O\right)\left(c\right)\\ +3{\mathrm{CO}}_{2}\left(g\right)+10H_{2}O\left(l\right)\to {\mathrm{Al}}_{2}O_{3}\cdot 3H_{2}O\left(c\right)\\ +2{\mathrm{CaCO}}_{3}\left(c\right)+3\mathrm{CaO}\cdot {\mathrm{Al}}_{2}O_{3}\cdot {\mathrm{CaCO}}_{3}\cdot 11H_{2}O\left(c\right)\\ +3\mathrm{CaO}\cdot {\mathrm{Al}}_{2}O_{3}\cdot 3{\mathrm{CaSO}}_{4}\cdot 32H_{2}O\left(c\right) \end{array}$$ (10)

The X-ray diffraction evidence includes a broadening of the peaks for the moist samples in the neighborhood of 9.0 and 8.0 A until they extended into the 7.5–7.8 A region, together with the appearance of a peak in the 9.5–10.0 A region. In the sample with the highest water content (about 20 “H_2_O”) the broad area separated into a distinct peak at 7.65 A, and a peak at 3.80 A was also evident. This trend is suggestive of the appearance of calcium aluminate monocarbonate, 3CaO·Al_2_O_3_·CaCO_3_·11H_2_O.

The 20 “H_2_O” sample showed the peaks of calcium aluminate trisulfate at 9.8, 5.6, 4.7, 3.9, and 2.8 A. Although wet samples of the monosulfate show the first three peaks, it is possible to distinguish the two types of pattern by means of the relative peak intensities, as pointed out in section 2.2. It is apparent that the trisulfate was being formed during the exposure.

Chemical tests showed a pickup of 29 percent of the original weight in a sample exposed to 100 percent relative humidity for 6 months. Of this weight increase, 11.9 percent was CO_2_ and 17.1 percent was H_2_O, quantities whose ratio is close to the H_2_O: CO_2_ ratio to be expected from eq (10), but which are greater in magnitude than the total weight pickup (17%) to be expected if that reaction went to completion. The pickup of CO_2_ is definitely established.

The acid solutions obtained in the calorimeter from these exposed samples were turbid. The undissolved material was apparently hydrated alumina. It dissolved slowly in the acid during the determination, liberating heat slowly and producing a fictitious final rating period and an incorrect calculated thermal-leakage constant for the determination.

In order to obtain an accurate estimate of the heat of solution of these exposed samples, it was necessary to use the minute-to-minute heat-leakage correction described briefly in reference [5]. The initial rating period, the known heat-leakage constant of the calorimeter, and the initial temperature gradient between calorimeter and bath were used to determine the stirring energy. The total heat leakage was then determined for each 1- or 2-min reading interval by adding the stirring energy to the product of the heat-leakage constant and bath-calorimeter gradient for that interval. By subtracting the total heat leakage thus calculated from the observed temperature rise for the interval, a minute-to-minute record of the corrected temperature rise was obtained.

**Figure 4:**
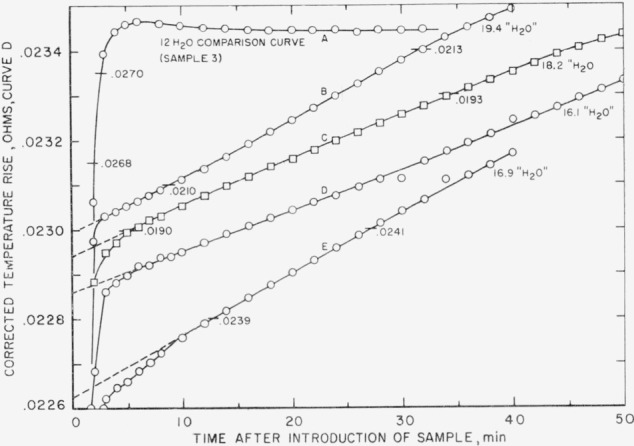
Temperature rise in calorimeter (in ohms) versus time after introduction of sample, for samples conditioned at 100 percent relative humidity A similar curve for an unexposed sample is shown for comparison. All curves are plotted to the same scale, but their ordinates represent different ranges of temperature rise. Ordinates at left are for curve D. Two ordinates are labeled or each of the other curves. Curve A: Sample 3, conditioned at 33 percent relative humidity, shown for comparison. Curve B: Sample 7, conditioned at 100 percent relative humidity to 19.4 “H_2_O”. Curve C: Sample 7, similarly conditioned to 18.2 “H_2_O”. Curve D: Sample 7, similarly conditioned to 16.1 “H_2_O”. Curve E: Sample 7, similarly conditioned to 16.9 “H_2_O”.

Figure 4 shows the trend of the corrected temperature rise for the exposed samples. For comparison, a similar plot for one of the 33 percent relative-humidity samples is shown, curve A. Note that curve A settles down to a constant value for the corrected temperature rise after 12 min whereas the curves for the exposed samples show a rapid rise (representing solution of the aluminate sulfates) followed by a slower, almost linear rise (representing slow solution of the hydrated alumina). By extrapolating the slow-rise portion of the curve to zero time, the heat of solution of the rapidly dissolving constituents of the sample was obtained.

It should be noted that thermal equilibrium in the calorimeter is normally reached within about 5–6 min, whereas curve A becomes horizontal only after 12 min. The small drop in the curve between 7 and 12 min is not believed to represent a temperature lag. It is considered rather to reflect non equilibrium endothermic release of CO_2_ gas derived from solution of the small quantity of CaCO_3_ in sample 3, as discussed in detail in reference [5]. A similar curve for sample 5, which was calculated to contain both CaCO_3_ and A1_2_O_3_·3H_2_O, shows in succession a rapid temperature rise, a fall (CaCO_3_), and finally a protracted rise (Al_2_O_3_·3H_2_O). This behavior of the temperature rise—time curve serves in several instances to confirm the impurities suggested by chemical analysis.

Calorimetric evidence is consistent with the reaction of eq (10) although it is not conclusive evidence of the reaction. Simple hydration of the monosulfate or physical water pickup would produce a curve either continuous with the 8–12H_2_O curve or higher than the right-hand curve of figure 3. If complete chemical reaction with H_2_O alone had occurred, with transformation to the trisulfate, hydrated alumina, and Ca(OH)_2_, the corresponding heat of solution of the mixture could not have been lower than 132 cal/g. The exposed samples, however, had heats of solution ranging from 150 to 115 cal/g. If eq (10) had gone to completion, the heat of solution would have been 99 cal/g. The calorimetric results are thus consistent with eq (10) provided the samples did not decompose completely during the exposure of 2 months or less. To reconcile the experimental heats of solution completely with the calculated values, it is necessary to assume that some of the weight picked up by the samples is sorbed H_2_O. Calculations based on these heats of solution indicate further that a maximum of 70 percent conversion of the monosulfate to the products of eq (10) was approached during the 2-month period.

Further chemical evidence is apparent in the results of exposure to 100 percent relative humidity of a portion of sample 1 originally intended for a loss-on-ignition determination. After exposure to the moist air at several temperatures, the sample was reconditioned to constant weight at 33 percent relative humidity. Its loss on ignition at 1,000 °C after this treatment was 41.3 percent as compared with the original value of 34.5 percent. A sample of monosulfate completely converted according to eq (10) would have a loss on ignition of 44 percent; one 70 percent converted, 41 percent. In view of the speculative nature of this discussion, the close agreement between the experimental and calculated loss should be viewed with caution, but the similarity is nevertheless encouraging.

It is apparently difficult to protect samples of calcium aluminate monosulfate from CO_2_ pickup in a moist atmosphere. Kantro, Copeland, and Anderson [9] report the same experience. A new approach is being made to the thermochemistry of samples of the compound containing more than 12H_2_O, by conditioning fresh wet pastes at 79 percent relative humidity (over saturated NH_4_Cl). Wet pastes previously exposed at 100 percent relative humidity were nonuniform and difficult to handle in the calorimeter. It is expected that results based on this approach will be reported in a later publication.

## 5. Summary

Heats of formation of 3CaO·Al_2_O_3_·CaSO_4_·12H_2_O(c), and of less completely hydrated samples of this compound ranging from 8 to 12H_2_O, have been determined by the heat-of-solution method, with 2*N* HCl as the solvent. Heats of reaction have been determined for the formation of 3CaO·Al_2_O_3_·CaSO_4_·12H_2_O(c) from 3CaO·A1_2_O_3_·6H_2_O(c), CaSO_4_·2H_2_O(c), and H_2_O(1), and for the formation of 3CaO·Al_2_O_3_· 3CaSO_4_·31–32H_2_O(c) from 3CaO·Al_2_O_3_ CaSO_4_·12H_2_O (c), CaSO_4_·2H_2_O(c), and H_2_O(1).

The heat of solution of 3CaO·Al_2_O_3_·CaSO_4_·*n*H_2_O in *2N* HCl, in kcal/mole, may be expressed over the 8–12H_2_O range by a quadratic formula:

$$-\Delta H=214.664-14.096n+0.5069n^{2}.$$

The change of the heat of solution with H_2_O content may be calculated from the expression:

$$-\frac{d\left(\Delta H\right)}{dn}=1.0139n-14.096$$

from which the rate at which the heat of solution of the 12H_2_O hydrate varies with a small change in water content is

$$-\frac{d\left(\Delta H\right)}{dn}=-1.93\;\text{kcal/mole per mole}\;H_{2}O.$$

Calcium aluminate monosulfate is a metastable compound and can be formed from solution only within certain limits of the concentration ratios of the various reacting ions. If prepared and filtered within about 7 hr, a CaO/Al_2_O_3_ molar ratio of not less than 9 is necessary to prevent conversion to the trisulfate. The minimum CaO/Al_2_O_3_ molar ratio is significant because a high OH^−^ concentration favors formation and longer persistence of the monosulfate, and all or most of the CaO is obtained from Ca(OH)_2_; from the mass-action law alone, a high CaO/Al_2_O_3_ ratio would be expected to produce the reverse effect.

In the presence of moisture and CO_2_, evidence points to the probability that the monosulfate is converted to calcium aluminate trisulfate, calcium aluminate monocarbonate, calcium carbonate, and hydrated alumina. Although the evidence is not conclusive, an equation is suggested for this transformation.

## Figures and Tables

**Figure 5:**
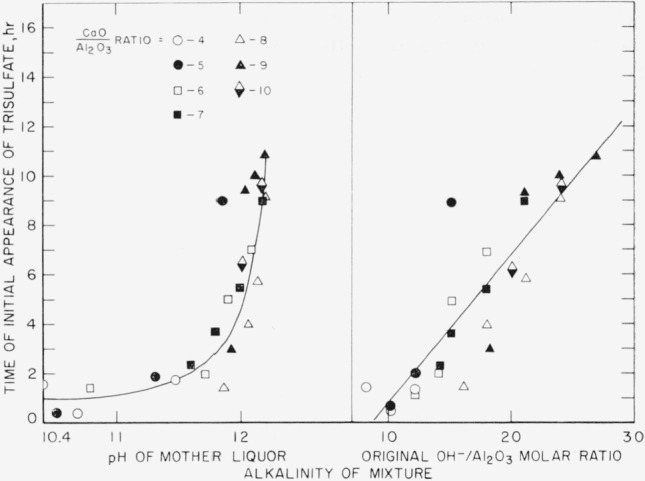
Effect of alkalinity on time required for initial conversion of calcium aluminate monosulfate to calcium aluminate trisulfate.

**Table 1:** Properties of the original preparations of calcium aluminate sulfates

Sample designation	1	2	3	4	5	6	7	8	9	10	11
											
Original concentration of mixture:											
CaO g/liter	1.088	1.047	1.051	0.983	1.025	(a)	(a)	(a)	0.914	……	……
Al_2_O_3_ g/liter	.114	.226	.107	.075	.207	(a)	(a)	(a)	.253	……	……
SO_3_ g/liter	.239	.380	.225	.315	.358	(a)	(a)	(a)	.469	……	……
Molar ratio:											
CaO: Al_2_O_3_	17.4	8.5	17.9	23.9	9.0	a 19.7	a 12.9	a 12.5	6.6	18.3	20.2
SO: Al_2_O_3_	2.7	2.2	2.7	5.4	2.2	2.9	2.9	2.9	2.4	3.0	3.0
OH^−^: Al_2_O_3_	29.4	12.7	30.4	37.1	13.6	39.5	25.8	25.0	8.4	(b)	40.4
Final concentration of solution:											
CaO g/liter	.850	.562	.846	.855	.624	a .910–.937	a .801–.811	……	.247	present	present
Al_2_O_3_ g/liter	.001	.000	.000	.001	.001	.000	.000	……	.058	absent	c absent
SO_3_ g/liter	.168	.215	.182	.313	.253	a .144–.169	a .219–.253	……	.013	c absent	c present
Composition of product:											
Weight percent:											
CaO	36.03	36.15± .01	35.98± .03	35.48± .21	35.70± .06	36.20± .004	35.99± .02	37.35± .11	……	……	……
Al_2_O_3_	16.40	16.76± .05	16.13± .02	16.14± .02	16.08± .01	16.35± .01	15.90± .02	16.88± .11	……	……	……
SO_3_	12.74	12.64± .11	12.65± .002	12.55± .02	13.07± .10	12.93± .02	13.56± .01	13.24± .11			
CO_2_	.31±.02	.09± .004	.45± .004	(d)	.23± .01	.30± .000	.24± .01	.41± .000	……	……	……
H_2_O	e 34.52	34.19	34.77± .07	(d)	34.93± .03	34.25± .05	34.36± .04	32.02± .03	……	……	……
Molar ratio:											
CaO: Al_2_O_3_	3.99_4_	3.92_1_± .01_1_	4.05_6_± .00_5_	3.99_5_± .02_4_	3.89_9_± .00_7_	4.02_5_± .00_0_	4.11_4_± .00_5_	4.02_3_± .02_8_	f 6.2	……	……
SO_3_ : Al_2_O_3_	.98_9_	.96_0_± .00_9_	.99_9_± .00_1_	.99_0_± .00_1_	1.03_5_± .00_8_	1.00_7_± .00_1_	1.08_6_± .00_4_	0.99_9_± .01_0_	f2.9		
CO_2_:Al_2_O_3_	.04_4_	.01_2_± .00_1_	.06_4_± .00_5_	(d)	.03_3_± .00_1_	.043± .00_0_	.03_5_± .00_2_	.06_0_± .00_1_			
H_2_O: Al_2_O_3_	11.912	11.545	12.20_0_± .02_8_	(d)	12.37_6_± .01_4_	11.85_6_± .01_9_	12.22_8_± .02_0_	10.73_4_± .07_1_	……	……	……
Molar ratios of compounds present, to Al_2_O_3_:											
3CaO·Al_2_O_3_· CaS0_4_·*n*H_2_O	.98_9_	.96_0_± .00_9_	.99_9_± .00_1_	.99_0_± .00_1_	.89_8_± .00_8_	1.00_0_± .00_0_	1.00_0_ ± .00_0_	.99_9_± .01_0_			(g)
where *n* =	12.01_1_	11.82_8_	12.21_2_± .02_8_	d12.54	h12	11.84_2_± .02_0_	12.05_6_± .02_1_	10.74_2_± .15_5_	……	……	……
3CaO·Al_2_O_3_·3CaSO_4_·31H_2_O	……	……	……	……	.04_6_± .00_5_	……	……	……	(i)	(i)	
CaCO_3_	.03_8_	.01_2_± .00_1_	.06_0_± .00_6_	d.03	.03_3_± .00_1_	.01_8_ ± 00_1_	.02_8_± .00_6_	.02_3_+ .02_8_ – 023			
Ca(OH)_2_		.06_9_± .01_8_	……	……	……	……	……	……	……	……	……
CaSO_4_·2H_2_O	……	……	……	……	……	.00_7_ ± .00_1_	.08_6_± .00_4_	……	……	……	……
Al_2_O_3_·3H_2_O	.01_1_	.04_0_± .00_9_	……	.01_0_ ±.00_1_	.05_6_± .03_5_	……	……	.00_1_± .01_0_ – .00_1_	……	……	……
CO_2_	.00_6_	……	……	d .01	……	.02_5_± .00_1_	.00_7_± .00_6_	.03_7_± .02_8_	……	……	……
Heat of solution of the sample:											
−ΔH, (from table 5)_cal/g	190.14	194.64	186.66	185.02	186.44	192.42	185.30	201.61	……	……	……
standard error cal/g±		.21	.19	……	.12	.12	.03	……	……	……	……
Corrected heat of solution of the 3CaO·Al_2_03·CaSO_4_·*n*H_2_O											
−ΔH, cal/g kcal/mole..	190.5_1_ 118.6_3_	191.4_4_ 118.5_8_	187.6_5_ 117.5_3_	185.2_9_ 117.1_5_	197.7_3_ 123.0_9_	193.5_2_ 119.9_2_	191.0_2_ 119.0_9_	202.5_8_ 121.5_2_	…… ……	…… ……	…… ……

a 5-batch mixtures; concentrations variable. Molar ratios given are overall, for all 5 batches.

b CaO dissolved in sucrose.

c Absence of ${\mathrm{SO}}_{4}^{-}$ in final solution is consistent with formation of 3CaO·Al_2_O_3_·3CaS0_4_·*m*H_2_O; presence of ${\mathrm{SO}}_{4}^{-}$ and simultaneous absence of Al_2_O_3_ is consistent with formation of 3CaO·Al_2_O3·CaSO_4_·*n*H_2_O.

d Not determined because of insufficient sample. H_2_O and CO_2_ estimated.

e Calculated by difference. Insufficient sample for determination.

f Calculated from initial concentration of mixture and final concentration of solution.

g X-ray diffraction pattern showed peaks of 3CaO·Al_2_O_3_·CaSO_4_·*n*H_2_O.

h Assumed to be 12H_2_O.

i X-ray diffraction pattern showed peaks of 3CaO·Al_2_O_3_·3CaSO_4_·*n*H_2_O.

**Table 2:** X-ray diffraction patterns of the calcium aluminate sulfates

Preparation		Principal identifying lines								Pattern classification^a^
Designation	Conditioned at relative humidity	9.5–10.0A	8.8–9.3A	8.0–8.2A	7.5–7.8A	5.7A	4.7–4.9A	4.4–4.6A	3.8–4.1A	
										
	*Percent*									
2	33	……	b 5	4	……	……	……	4	3	Monosulfate Bc.
3	33	……	5	……	……	……	……	4	2½	Monosulfate B.
4	c33	……	5	3	……	……	……	4	2	Monosulfate Bc.
5	e33		5	3				5	2	Monosulfate Bc.
	d33	……	4	4	……	……	……	2½	2½	Monosulfate bc.
	e33	……	4	5	……	……	……	2	3	Monosulfate bC.
6	wet	5	……	……	……	2	5	……	2	Monosulfate A.
	c33	……	5	3	……	……	……	4	3	Monosulfate Bc.
	d33		5	3				4	2½	Monosulfate Bc.
	0			5			1	1	5	Monosulfate C.
7	Wet	5	……	……	……	2	5	……	2	Monosulfate A.
	c33	……	5	3	……	……	……	4	3	Monosulfate Bc.
	d33		3	5			1	2	3	Monosulfate bC.
	f100 (16 “H_2_O”) f 100 (17 “H_2_O”) f 100 (20 “H_2_O”)	4 4 4	5 5	4 4 2	4 4 5	3 3 3	1 1 1	4 4 1	2 2 2	} Monosulfate Bc, trisulfate and monocarbollate.
	12	3	5	5	……	2	2	4	5	Monosulfate bC.
	5	1	……	5	……	2	2	2	5	Monosulfate C.
9	Wet	5	1	……	……	5	4	……	5	Trisulfate.
10		5	1	……	……	5	4	……	5	Trisulfate.
11	Wet	5	……	……	……	……	3	……	1	Monosulfatc A.

a Letters refer to type of monosulfate pattern. Upper-case type indicates predominant pattern.
wet type of calcium aluminate monosulfate. high-humidity type. low-humidity type.

b Numbers refer to relative intensity of lines.
5—very strong. 4—strong. 3—medium. 2—weak. 1—very weak.

c Exposed to X-ray immediately after removal from conditioning chamber; pattern completed in 10 min.

d Exposed to X-ray some time after removal from conditioning chamber, but protected from atmosphere; pattern completed in 10 min.

e Same as d, but pattern completed in 40 min, following the 10-min pattern.

f 33 percent R.H.-conditioned samples, exposed to 100 percent R.H., for various lengths of time.

**Table 3:** Properties of the dried preparations of calcium aluminate monosulfate

Conditioning agent	Saturated LiCl	23 *N* H_2_SO_4_				CaO		
			
Relative humidity %	12	5	5	5	5	0	0	0
Original sample exposed	7	2	6	7	8	6	7	8
Time exposed days	103	22	25	95	25	21	14	25
H_2_O content, *n*, of the 3Ca0·Al_2_O_3_·CaS0_4_·*n*H_2_O	11.83_7_	10.67_8_	10.66_5_	10.56_8_	10.62_2_	8.77_1_	8.60_7_	8.61_5_
Heat of solution of the sample, −Δ*H* cal/g	185.84	203.71	203.78	198.38	202.38	229.17	228.74	229.83
Corrected heat of solution of the 3CaO·Al_2_O_3_·CaSO_4_·*n*H_2_O,−Δ*H* cal/g	191.6_1_	203.0_7_	204.9_9_	204.7_5_	203.3_6_	230.4_8_	235.2_2_	231.0_7_
kcal/mole	118.7_2_	121.5_8_	122.6_8_	122.1_8_	121.5_5_	130.0_7_	132.0_4_	129.7_6_

**Table 4:** Calibration of calorimeter with 740 grams *2*N *HCl*

Run	Time	*I*	*E*	*Q*	Δ*R_c_*	Heat capacity
						
	*sec*	*amp*	*Volts*	*j*	*Ohm*	*j/ohm*
1	839.993	0.240711	16.6215	3360.793	0.117847	a 28, 518.2_7_
2	809.993	.238626	16.4756	3184.493	.111660	28, 519.5_5_
3	926.993	.237635	16.4076	3614.364	.126718	28, 522.8_9_
4	931.993	.234793	16.2123	3347.664	.124471	28, 501.9_3_
5	899.993	.240531	16.6090	3595.453	.126230	28.483.3_2_
6	839.993	.241842	16.7005	3392.633	.119156	28.472.2_0_
7	839.993	.240185	16.5849	3346.065	.117589	28, 455.6_0_
8	810.993	.246121	16.9946	3392.163	.119179	28.462.7_6_
	
Mean heat capacity j/ohm..						b 28, 492.0_6_±9.59
Mean heat capacity cal/ohm..						6, 889.7_7_±2.29
	
1	1499.993	0.096368	6.65251	961.629	0.033684	a 28, 548.5_4_
2	1379.993	.096579	6.66773	888.669	.031169	28, 511.3_1_
3	1259.993	.096179	6.640233	804.697	.028218	28, 517.1_5_
4	899.993	.112554	7.77112	787.197	.027636	28, 484.4_8_
5	959.993	.112523	7.76940	839.261	.029422	28, 524.9_5_
6	910.493	.112549	7.77086	796.319	.027908	28, 533.7_2_
7	959.493	.112450	6.65251	838.128	.029442	28, 467.0_9_
	
Mean heat capacity j/ohm..						c 28, 512.4_7_±10.66
Mean heat capacity cal/ohm..						6, 814.6_4_±2.55

a It is not the authors’ intention to imply that the heat-capacity values are either precise or accurate to the number of figures tabulated. These figures are carried through the calculations to facilitate the estimation of standard error. The heat-of-sclution values obtained from them in tables 5 and 6 are rounded off.

b Long stirrer used. Temperature rise approximately 1 °C. Calibration used for determining heat of solution of samples 1 and 2.

c Short stirrer used. Temperature rise approximately 0.3 °C. Calibration used for determining heat of solution of samples 3, 4, 5, 6, 7, 8. and all dried and moist samples.

**Table 5:** Details of heat-of-solution determinations on samples of calcium aluminate monosulfate

Sample	Run	Corrected rise	Sample weight	Heat of solution, *−*Δ*H*					
				Uncorrected	Correction for heat capacity of sample	Corrected	Std. error	Corrected	Std. error
									
		*ohm*	*g*	*j*/*g*	*j*/*g*	*j*/*g*	*j*/*g*	*cal*/*g*	*cal*/*g*
1^a^	…	0.026628	0.9536	795.60	−0.07	795.53	……	190.14	……
									
2^a^	1	0.026372	0.9210	815.84	−0.26	815.58	……	……	……
	2	.028594	1.0026	812.59	+.12	812.71	……	……	……
	3	.029623	1.0353	815.24	−.38	814.86	……	……	……
				
Mean, before correction for impurities						814.38	±0.86	194.64	±0.21
				
3^b^	1	0.027118	0.9889	781.88	−0.10	781.78	……	……	……
	2	.026801	.9792	780.39	−.20	780.19	……	……	……
				
Mean, before correction for impurities						780.98	±.79	186.66	±.19
				
4^b^	…	0.027587	1.0158	774.34	−0.23	774.11	……	185.02	……
									
5^b^	1	0.018405	0.6723	780.56	−0.20	780.36	……	……	……
	2	.030042	1.0970	780.83	−.28	780.55	……	……	……
	3	.027581	1.0101	778.54	+.04	778.58	……	……	……
	4	.027295	0.9966	780.90	−.06	780.84	……	……	……
				
Mean, before correction for impurities						780.08	±.51	186.44	±.12
				
6^b^	1	0.028732	1.0143	807.67	−0.92	806.75	……	……	……
	2	.028464	1.0067	806.18	−.85	805.33	……	……	……
	3	.028248	1.0003	805.18	−.59	804.59	……	……	……
	4	.028277	1.0033	803.60	−.07	803.53	……	……	……
	5	.028342	1.0036	805.20	+.01	805.21	……	……	……
				
Mean, before correction for impurities						805.08	±.52	192.42	±.12
				
7^b^	1	0.027108	0.9973	775.01	−0.03	774.98	……	……	……
	2	.027173	.9990	775.54	−.19	775.35	……	……	……
	3	.027310	1.0035	775.96	−.36	775.60	……	……	……
	4	.027494	1.0105	775.78	−.60	775.18	……	……	……
				
Mean, before correction for impurities						775.28	±.13	185.30	±.03
				
8^b^	…	0.029173	0.9870	842.75	+0.80	843.55	……	201.61	……
									
6 (0% R.H.)^b^	…	0.030891	0.9179	959.56	−0.70	958.86	……	229.17	……
7 (0% R.H.)^b^	…	.029708	.8845	957.66	−.62	957.04	……	228.74	……
8 (0% R.H.)^b^	…	.032505	.9636	961.81	−.19	961.62	……	229.83	……
2 (5% R.H.)^b^	…	.024698	.8256	852.96	−.65	852.31	……	203.71	……
6 (5% R.H.)^b^	…	.028828	.9640	852.65	−.03	852.62	……	203.78	……
7 (5% R.H.)^b^	…	.025596	.8786	830.65	−.64	830.01	……	198.38	……
8 (5% R.H.)^b^	…	.028965	.9761	846.09	+.65	846.74	……	202.38	……
									
7 (12% R.H.)^b^	1	0.027792	1.0173	778.94	−0.75	778.19	……	……	……
	2	.027029	.9913	777.43	−.53	776.90	……	……	……
				
Mean, before correction for impurities						777.54	±.65	185.84	±.15
				
7 (100% R.H.)^b^	…				……	……	……		……
16.1 “H_2_O”^c^	…	0.02286	1.0450	623.7	……	……	……	d 149.1	……
16.9 “H_2_O”^c^	…	.02372	1.1483	589.0	……	……	……	d 140.8	……
18.1 “H_2_O”^c^	…	.01894	0.9910	544.9	……	……	……	d 130.2	……
19.4 “H_2_O”^c^	…	.02089	1.1918	499.8	……	……	……	d 119.4	……
20.4 “H_2_O”^c^	…	.02084	1.2398	479.3	……	……	……	b 114.6	……

a Heat capacity of calorimeter 28,492.0_6_ joules/ohm.

b Heat capacity of calorimeter 28,512.4_7_ joules/ohm.

c See text for discussion of the composition of these samples.

d No correction was made for the heat capacity of these samples, because the sample temperature could not be measured in the short time between removal from conditioning chamber and introduction into calorimeter.

**Table 6:** Heat-of-solution determinations on *CaS0_4_·2H_2_0* for different sample-acid ratios

Sample weight	Acid weight	Corrected rise	Heat capacity of calorimeter	Heat of solution, −Δ*H*					
				Uncorrected	Corrected for heat capacity of sample	Corrected	Std. error	Corrected	Std. error
									
G	g	Ohm	j/ohm	j/g	j/g	j/g	j/g	eal/g	cal/g
0.2768	740.0	−0.001370	28, 512.4_7_	−141.12	−0.57	−141.69	……	……	……
.2831	……	−.001372	……	−138.18	−.84	−139.02	……	……	……
.2863	……	−.001438	……	−143.21	−.44	−143.65	……	……	……
				
Mean						−141.45	±1.34	−33.81	±0.32
				
a 0.4340	600.0	−0.002398	b 24, 479.9_0_	−135.26	+0.19	−135.07	……	……	……
a.4285	……	−.002412	……	−137.80	+.09	−137.71	……	……	……
a.4239	……	−.002431	……	−140.39	+.10	−140.29	……	……	……
				
Mean						−137.69	±1.51	−32.91	±0.36
				
a 1.0171	600.0	−0.005708	b 24, 479.9_0_	−137.38	+0.02	−137.36	……	……	……
a 1.0060	……	−.005726	……	−139.34	+.03	−139.31	……	……	……
a 1.0205	……	−.005742	……	−137.74	−.01	−137.75	……	……	……
				
Mean						−138.14	±0.59	−33.02	±0.14

a These determinations were reported in abbreviated form in reference [6].

b See reference [5] for calibration details.

**Table 7:** Thermodynamic properties of compositions of calcium aluminate monosulfate, for different water contents

Moles H_2_O per mole, *n*	8	9	10	11	12
					
Heat of solution in 2*N* HCl of 3CaO·Al_2_O_3_·CaSO_4_·*n*H_2_O					
Δ*H* kcal/mole	−134.3_4_	−128.8_6_	−124.3_9_	−120.9_4_	−118.5_1_
Heat of formation from 3CaO·Al_2_O_3_·6H_2_O(c), CaSO_4_·2H_2_O(c), and H_2_O(1)					
Δ*H* kcal/mole	+0.92	−4.5_8_	−9.0_6_	−12.5_3_	−14.9_8_
Heat of formation from the elements,					
Δ*H°_f_* kcal/mole	−1811._1_	−1885._0_	−1957._7_	−2029._5_	−2100._3_

## Footnotes

1 Figures in brackets indicate the literature references at the end of this paper.

2 Standard error, formerly referred to as standard deviation of the mean,

$$=\sqrt{\frac{\Sigma x_{i}^{2}-\frac{{\left(\Sigma x_{i}\right)}^{2}}{n}}{n\left(n-1\right)}}$$

where *x*_i_ is the result obtained for one measurement, and *n* is the number 0 measurements.

3 Reactants for the trisulfate are the same as for the monosulfate. Reactants for the monocarbonate are CaCO_3_(c), 3CaO·Al_2_O_3_·6H_2_O(c), and H_2_O(1).

## 6. Appendix. Conditions for the Formation and Stability of Calcium Aluminate Monosulfate

### 6.1. Effect of Reactant Concentrations

The CaO—SO_3_—A1_2_O_3_—H_2_O system has been studied by Jones [16], D’Ans and Eick [17], and Eitel [18], who prepared equilibrium diagrams for two sets of products, a stable system in which the trisulfate is formed, and a metastable system in which the monosulfate is formed. However, detailed information on the conditions for forming the products of one or the other system is not generally available.

The concentration ratios necessary to produce either trisulfate or monosulfate within a given time limit bear no apparent relation to the stoichiometric ratios of the oxides in these compounds. Although the trisulfate exhibits a higher CaO/Al_2_O_3_ molar ratio (6:1) and a higher SO_3_/A1_2_O_3_ ratio (3:1) than the monosulfate (4:1 and 1:1 respectively), the monosulfate was formed most readily in this work when the original mixture had a CaO/Al_2_O_3_ ratio greater than about 8, for SO_3_/Al_2_O_3_ ratios ranging from 2.2 to 5.4 (see table 1). For CaO/Al_2_O_3_ ratios less than 8 or greater than about 50, the precipitate formed directly was either entirely or partly trisulfate. For CaO/Al_2_O_3_ ratios less than 6, a mixture of trisulfate and hydrated alumina tends to precipitate [2]. Unpublished work performed in this laboratory by Stearns [19] showed that, for SO_3_/A1_2_O_3_ molar ratios between 2 and 3, the most stoichiometrically accurate precipitates of monosulfate were obtained at CaO/Al_2_O_3_ ratios of 20 to 24.

The apparent contradiction in the fact that formation of the complex sulfate with the lower CaO/Al_2_O_3_ ratio requires an original mixture of higher ratio may be resolved if it is considered that the source of the CaO is primarily calcium hydroxide. The preparations with the most Ca(OH)_2_ had the highest OH^−^/Al_2_O_3_ ratios in the original mixture, and would be expected to favor formation of a more basic precipitate, that is, the aluminate monosulfate in preference to the trisulfate. The viewpoint was tested by preparing a series of small-scale batches at several CaO/Al_2_O_3_ ratios, with varying additions of NaOH such that the OH^−^/Al_2_O_3_ ratio was made to vary without corresponding changes in the CaO/Al_2_O_3_ ratio. Figure 5 shows the effect of OH^−^/Al_2_O_3_ ratio (as calculated from concentrations in the original mixture) on the time required to initiate conversion of the monosulfate to the trisulfate, as well as the dependence of this time on the of the mother liquor. At any one CaO/Al_2_O_3_ ratio from 4 to 10, the time at which the trisulfate first appeared increased as the OH^−^/Al_2_O_3_ ratio increased. For a constant OH^−^/Al_2_O_3_ ratio, the CaO/Al_2_O_3_ ratio did not affect the time consistently, but in most instances higher CaO/Al_2_O_3_ ratio accelerated the conversion to the trisulfate, in accordance with the effect to be expected from the law of mass action. The conclusions of Lerch, Ashton, and Bogue [1] on the role of alkalinity are thus confirmed.

With extremely high CaO/Al_2_O_3_ ratios, the mass-action effect of the Ca^++^ ion may be stronger than the influence of OH^−^, and trisulfate may be very readily precipitated at very high Ca^++^ concentrations.

The experience of other workers, who have been unable to prepare the monosulfate at room temperature, is possibly explained by these concentration-ratio requirements. For example, Kelly [20] attempted to prepare the monosulfate at room temperature from one liter of saturated CaSO_4_ solution containing 3.06 g of Al_2_(SO_4_)_3_·18H_2_O and two liters of saturated Ca(OH)_2_ solution. This mixture represents a CaO/Al_2_O_3_ molar ratio of 12, and an OH^_^/Al_2_O_3_ ratio of 18. Although this is within the range of possible monosulfate formation based on consideration of the OH^−^/Al_2_O_3_ ratio, it is near the low end of this range, and the OH^−^/SO_3_ ratio of Kelly’s mixture was 3. In all the samples prepared in this work, the OH^−^/SO_3_ ratio was not less than 6, except for the preparation which turned out to be entirely trisulfate, sample 9, in which the OH^−^/SO_3_ ratio was 3.5.

### 6.2. Procedure for Preparation of Large Batches

Preparations 1, 2, 3, 4, and 5 were made from saturated Ca(OH)_2_ solution, saturated CaSO_4_ solution, and calcium aluminate solution. The calcium aluminate solution was prepared by shaking high-alumina cement (previously ignited at 1,000 °C to oxidize sulfides) with CO_2_-free water for about 2.5 to 3 hr and rapidly filtering the supernatant liquid. (The calcium aluminate solution is metastable, and a precipitate appears unless it is used within a short time; if filtration is too slow, precipitation will occur during the filtration, but, once filtered, the solution is stable for several hours.) The calcium aluminate solution was added to the Ca(OH)_2_ solution in a 12-liter flask while stirring; calcium sulfate solution was then added, and the entire mixture was stirred about 2 hr. The mixture was then filtered through a Buchner fritted-glass funnel, drained as free as possible of mother liquor by suction, and transferred to a desiccator for conditioning to the desired H_2_O content.

As a result of the limited range of concentrations in which the monosulfate precipitate may be preserved during the time required for the operations, the yield of the compound is low for a single batch prepared as described. The low solubility of Ca(OH)_2_, 0.02 moles per liter, and the large CaO/Al_2_O_3_ molar ratio required, limits the quantity of Al_2_O_3_ that can be introduced, with the result that an individual 12-liter batch cannot theoretically yield more than 16 g of 3CaO·A.l_2_O_3_·CaSO_4_·12H_2_O under the most favorable conditions.

The yield cannot be increased by making several batches of the compound and mixing the filtered products because the precipitates obtained in different batches are slightly different in composition and the product, even if mixed, would not be uniform. The samples of the preparation taken for analysis might not have the same composition as the samples taken for calorimetry. The yield cannot be increased easily by increasing the volume of the mixture because excessively large reaction vessels would be needed. Potassium aluminate solution can be made more concentrated in A.l_2_O_3_ than calcium aluminate solution, but large reaction vessels would still be required to maintain the CaO/Al_2_O_3_ ratio.

The following technique was therefore resorted to for producing larger batches (preparations 6, 7, and 8). About 140 to 230 ml of reagent Al_2_(SO_4_)_3_ solution (0.08M) was added slowly to 11 liters of saturated Ca(OH)_2_ solution. The mixture was stirred 5 to 10 min., allowed to settle 5 to 10 min., and 5 to 9 liters of the supernatant liquid was drawn off and discarded. Calcium hydroxide and aluminum sulfate solutions were then added in the same proportion as in the first addition and in quantity sufficient to replace the mother liquor removed. After stirring 5 to 10 min. and allowing to settle, 5 to 9 liters of the supernatant liquid was again drawn off and discarded. Three more additions of Ca(OH)_2_ and A.l_2_(SO_4_)_3_ solutions were made by this alternate reaction-decantation technique. After the last addition, the entire remaining mass of precipitate produced was stirred for 15 min. and allowed to settle. Most of the supernatant liquid was withdrawn through the filter. The remainder was then stirred and the filtration completed. The total yield theoretically possible by this technique is about 40 g. The entire product is stirred at one time in mother liquor of the proper concentration. Analysis of the several mother-liquor batches showed no significant variations in concentration of CaO, but a steady increase in SO_3_ content consistent with the formation of a precipitate containing less than the original mixture. The multiple addition-decantation technique cannot he continued indefinitely because the precipitate must be filtered before conversion to the trisulfate can take place.
